# Unraveling the molecular basis of oxidative stress management in a drought tolerant rice genotype Nagina 22

**DOI:** 10.1186/s12864-016-3131-2

**Published:** 2016-10-04

**Authors:** Chandra Prakash, S. V. Amitha Mithra, Praveen K. Singh, T. Mohapatra, N. K. Singh

**Affiliations:** 1 ICAR-National Research Centre on Plant Biotechnology, Indian Agricultural Research Institute, Pusa Campus, New Delhi, 110 012 India; 2 Division of Seed Science and Technology, Indian Agricultural Research Institute, Pusa Campus, New Delhi, 110 012 India; 3 Indian Council of Agricultural Research, Krishi Bhavan, New Delhi, 110 001 India

**Keywords:** Drought tolerance, Functional polymorphism, Oxidative stress management, Nagina 22, Rice, Spikelet fertility

## Abstract

**Background:**

Drought stress tolerance for crop improvement is an important goal worldwide. Drought is a complex trait, and it is vital to understand the complex physiological, biochemical, and molecular mechanisms of drought tolerance to ﻿tackle﻿ it effectively. Osmotic adjustment, oxidative stress management (OSM), and cell membrane stability (CMS) are major components of cellular tolerance under drought stress. In the current study, we explored the molecular basis of OSM in the drought tolerant rice variety, Nagina 22 and compared it with the popular drought sensitive rice variety, IR 64, under drought imposed at the reproductive stage, to understand how the parental polymorphisms correlate with the superiority of Nagina 22 and tolerant bulk populations under drought.

**Results:**

We generated recombinant inbred lines (RIL) from contrasting parents Nagina 22 and IR 64 and focussed on spikelet fertility (SF), in terms of its correlation with OSM, which is an important component of drought tolerance in Nagina 22. Based on SF under drought stress and its correlations with other yield related traits, we used superoxide dismutase (SOD), glutathione reductase (GR), and ascorbate peroxidase (APX) activity assays to establish the relationship between SF and OSM genes in the tolerant and sensitive lines. Among the OSM enzymes studied, GR had a significant and positive correlation with single plant yield (SPY) under drought stress. GR was also positively correlated with APX but negatively so with SOD. Interestingly, none of the enzyme-morphology correlations were significant under irrigated control (IC). Through genome-wide SNP analysis of the 21 genes encoding for OSM enzymes, we identified the functional polymorphisms between the parents and identified superior alleles. By using network analysis of OSM genes in rice, we identified the genes that are central to the OSM network.

**Conclusions:**

From the biochemical and morphological data and the SNP analysis, the superiority of Nagina 22 in spikelet fertility under drought stress is because of its superior alleles for SOD (*SOD2, SODCC1, SODA*) and GR (*GRCP2*) rather than for APX, for which IR 64 had the superior allele (*APX8*). Nagina 22 can bypass *APX8* by directly interacting with *SODA*. For nine of the 11 genes present in the central network, Nagina 22 had the superior alleles. We propose that Nagina 22 tolerance could mainly be because of *SODA* which is a reactive oxygen scavenger in mitochondria which is directly associated with spikelet fertility.

**Electronic supplementary material:**

The online version of this article (doi:10.1186/s12864-016-3131-2) contains supplementary material, which is available to authorized users.

## Background

Plant photosynthesis occurs in specialized organelles known as chloroplast where visible light is absorbed mainly by the pigment molecules chlorophyll a, b and carotenoids [1]. The reaction centres of PSI and PSII present in chloroplast thylakoids are the major sites of reactive oxygen species (ROS), which are partially reduced forms of molecular oxygen; e.g. superoxide radical (O2^.-^), hydroxyl free radical (^.^OH), hydrogen peroxide (H_2_O_2_) and singlet oxygen, formed during electron transport activities in the chloroplasts, mitochondria and plasma membrane [2]. Photoreduction of oxygen to hydrogen peroxide (H_2_O_2_) in PSI was discovered in 1951 [3], and later, superoxide anion (O_2_^.^) was identified as the reduced product of molecular oxygen and superoxide dismutase (SOD), a key enzyme for the conversion of O_2_^.-^ to H_2_O_2_ and O_2_ [2]. H_2_O_2_ cannot accumulate in chloroplasts since it interferes with photosynthesis by rapidly oxidizing thiol-regulated enzymes [4], and therefore it is reduced to water by ascorbate peroxidases (APX) with the electrons derived from water splitting in PSII [5]. Glutathione reductase (GR), an NAD(P)H-dependent enzyme, effectively maintains a reduced pool of glutathione (GSH) levels inside the chloroplasts to prevent oxidative damage by ROS [6, 7]. Furthermore, in the ascorbate-glutathione pathway, APX and GR work synergistically to maintain ROS levels below the threshold. Catalases (CAT) convert H_2_O_2_ to water and molecular oxygen. These enzymes have extremely high maximum catalytic rates but low substrate affinities, and the reaction requires the simultaneous access of two H_2_O_2_ molecules in the active site [8, 9].

Disequilibrium between ROS production and antioxidant protection causes oxidative stress, and the accumulating ROS causes peroxidation of lipids, denaturation of proteins, mutation in DNA sequence and various other types of cellular oxidative damage [10–13]. Enhanced generation of ROS has been found in plants under a variety of abiotic stresses, such as salt, drought, chilling, heat, and metal or metalloid stresses [14–18].

Rice is one of the most widely consumed food crops, and is the most sensitive to drought stress during reproductive development [19–21], as rice spikelet fertility is extremely sensitive to water stress. In the absence of drought, yield potential determines grain production, but as drought becomes severe, drought escape or drought tolerance becomes important [22]. Spikelet fertility is directly related to yield and is known to be sensitive to water and heat stress in rice [23–25]. The upland rice variety, Nagina 22, is an internationally recognised hardy aus genotype frequently used in drought and heat tolerance studies [23, 24, 26]. It is reported to have comparatively less spikelet sterility under drought stress because of a more efficient antioxidant defence system for scavenging of H_2_O_2_ in panicles [27, 28].

Rice, besides being a staple food crop, is also a model crop for genomics, particularly in monocots, owing to its relatively smaller genome size and the availability of high quality genome sequences [29] and a wealth of genomic resources [30–32]. Numerous transcription factors and genes are implicated to be differentially expressed in rice under drought stress [33], and some of these genes been demonstrated to increase drought tolerance [34–36]. There have been multiple efforts to identify quantitative trait loci (QTLs) related to yield under drought tolerance and their subsequent introgression in major rice varieties [37–39]. Although complex traits such as yield are routinely dissected into their component traits namely grain size, test weight, and number of productive tillers per plant in rice [40], and functional markers have been developed [41, 42] for plant breeders, the same is not true in drought stress research.

Drought resistance can occur through either through drought avoidance (better root system, waxy leaves, etc.,) or other drought tolerance mechanisms such as better water use efficiency and cellular tolerance. Osmotic adjustment (OA), oxidative stress management (OSM), and cell membrane stability (CMS) are the major components of cellular drought tolerance. So far, only limited literature is available in rice in the detection of QTLs and their fine mapping and cloning of the causal genes associated with these physiological/biochemical traits related to drought tolerance. Some researchers have identified QTLs for CMS [43], osmotic adjustment [44–46], and water use efficiency [47] and all of these were coarse or preliminary mapping studies. However, the components associated with drought tolerance (OA, OSM and CMS) remain as associations for which neither the genetic basis nor the molecular basis has been unequivocally established. Since the enzymes governing OSM are well known, we decided to explore their role in spikelet sterility under drought stress in a mapping population of rice. This will validate any associations by using a genetical genomics approach.

In the present work, we have used a recombinant inbred population (RIL) derived from the genetic cross between the drought tolerant Nagina 22 and the rice mega variety IR 64, which is sensitive to drought stress at the reproductive stage. We have identified the extreme bulks for spikelet fertility under drought stress from this population and used them to investigate the mechanism of OSM.

## Methods

### Plant material and growth conditions

Rice genotypes, Nagina 22 (drought tolerant), IR 64 (drought sensitive) and their 281 RILs, (F_9_﻿_:10_ generation) were phenotyped for drought tolerance at the Experimental Farm, Indian Agricultural Research Institute (IARI), New Delhi (28°38′N and 77°12′E, 293 m elevation above sea level) during the 2011 and 2014 *Kharif* seasons. Nagina 22 was chosen as the tolerant parent because of its international importance and positive attributes such as better drought and heat tolerance [26]. IR 64 which is sensitive to drought stress at reproductive stage, was chosen as the other parent because it is a mega variety and regularly used by rice breeders for its improvement through marker- assisted backcross breeding [48]. Staggered nursery sowing at 5-days interval was done to synchronize the flowering time of RILs as closely as possible. This was done based on the flowering data of RILs recorded in the previous two generations (data not shown). The experiment was done in an augmented block design with check lines repeated after every 16 RILs. The check lines used were the rice cultivars Nagina 22 and IR 64 (parents) in both seasons. Lines were planted in plots of 2 m × 0.6 m with a spacing of 20 cm × 20 cm between hills and rows, both in irrigated and water stress conditions. In 2014, a subset of RILs (200) was planted in an augmented design under a rainout shelter at IARI in 3 m × 0.20 m plot size with 15 cm × 20 cm spacing. Standard cropping practices were adopted for proper nutrient and weed management. All plots were maintained at field capacity and water stress was imposed by withholding irrigation at the initiation of the booting stage to 10 days after anthesis in the majority of the lines. Phenotyping for physiological traits was done after the RILs showed leaf rolling and drying, whereas yield related parameters were measured at maturity.

On the basis of phenotype data recorded on spikelet sterility (SS), and days to 50 % flowering (DTF) under drought stress in the 2011 *Kharif*, 41 RILs were selected for validation of their SS phenotype. In 2014, these 41 RILs were carefully phenotyped in the field for SS and yield related traits under stress. From these data, two extreme groups termed ‘tolerant bulk’ (TB) and ‘susceptible bulk’ (SB) were selected. A third group, termed ‘random bulk’ (RB) was created from the rest of the RIL population using a random number method. Each group comprised of eight RILs and their flag leaves were collected and stored at −80 °C from both the irrigated and drought treatment plots. All the observations were individually measured in the lines constituting the bulks with adequate sampling (five plants per genotype) and averaged to obtain the trait means of the respective bulks.

### Observation of physiological and agronomic parameters

Chlorophyll content of flag leaves was measured using the SPAD-502, a portable chlorophyll meter, which measures the greenness or the relative chlorophyll concentration of leaves. Plant height, panicle number, tiller number, number of spikelets per panicle (SPP), and single plant yield (SPY) were measured from five plants per RIL. SS was calculated as ratio of unfilled spikelets to total spikelets from at least three main panicles and was expressed as percent (%). To assess yield reduction under stress compared to irrigated environments, the Stress Susceptibility index (SSI) was calculated using the formula, SSI = ^1-[(Yi)s/(Yi)ns]^/_1-[(Yps)/(Ypns)],_ where Yi, Yp, s, and Yns represent individual yield, population mean yield, stress condition, and non-stress conditions, respectively [49].

### Preparation of enzyme extracts

Enzyme extracts for superoxide dismutase (SOD; EC 1.15.1.1), Glutathione reductase (GR; EC 1.6.4.2), Ascorbate peroxidase (APX; EC 1.11.1.1) and Catalase (EC 1.11.7.6) was prepared from the flag leaf tissue samples that were pre-frozen in liquid nitrogen to prevent proteolytic activity, and ground in 3 ml extraction buffer containing 0.1 M phosphate buffer (pH 7.5) and 0.5 mM EDTA. Extracts were centrifuged for 20 min at 15,000 g and the supernatant was used in the enzyme assay [50]. The Bradford method was used to estimate protein concentrations of all the samples before performing enzyme assays. Each enzyme assay was scaled down to a 200 μl volume and all the spectrophotometric measurements were done in a 96 well plate reader (Varioskan™, Thermo Scientific,USA). Enzyme assays were performed using three biological and three technical replicates. For each enzyme assay, at least eight readings at an interval of 30 s were collected and simple regression analysis was performed on optical density (O.D) vs. time to calculate the change in absorbance per minute. Means of biological and technical replicates were collected to calculate final change in OD per minute. SOD activity in the samples was estimated by recording the decrease in the OD of nitro-blue tetrazolium dye by the enzyme [50]. A volume of 200 μl reaction mixture contained 13 mM methionine, 25 mM nitroblue tetrazolium chloride (NBT), 0.1 mM EDTA, 50 mM phosphate buffer (pH 7.8), 50 mM sodium carbonate and 10 μl enzyme extract. The reaction was started by adding 2 mM riboflavin and placing the tubes under two 15 W fluorescent lamps for 15 min. The reaction was stopped by switching off the light and keeping the tubes in the dark. A complete reaction mixture without enzyme, which gave the maximal colour, served as a positive control. A non-irradiated complete reaction mixture served as a blank. Separate controls that lacked enzymes were used for total SOD and inhibitor studies. The absorbance was recorded at 560 nm, and one unit of enzyme activity was taken as the amount of enzyme that reduced the absorbance reading to 50 % in comparison to control tubes. APX was assayed by recording the decrease in optical density because of ascorbic acid at 290 nm [51]. The 200 μl reaction mixture contained 50 mM potassium phosphate buffer (pH 7.0), 0.5 mM Ascorbic Acid, 0.1 mM EDTA, 1.5 mM H_2_O_2_ and 10 μl enzyme extract. The reaction was initiated with the addition of H_2_O_2_. The molar extinction coefficient of 2.8 mM^−1^ cm^−1^ was used for the calculation of enzyme activity. Absorbance was measured at 290 nm in a UV-visible spectrophotometer. GR was assayed by recording the increase in absorbance in the presence of oxidized glutathione and DTNB (5,5-dithiobis-2-nitrobenzoic acid) [52]. The 200 μl reaction mixture contained 200 mM potassium phosphate buffer (pH 7.5) containing 1 mM EDTA, 1.5 mM DTNB (5,5-dithiobis-2-nitrobenzoic acid) in 0.01 M potassium phosphate buffer (pH 7.5), 0.2 mM NADPH, 10 μl enzyme extract and distilled water to make up the final volume. The reaction was initiated by adding 0.2 mM GSSG (oxidized glutathione or glutathione disulphide). The increase in absorbance at 412 nm was recorded and 6.22 mM^−1^cm-^1^ was used as the molar extinction coefficient for calculation of enzyme activity. Catalase was assayed by measuring the disappearance of H_2_O_2_ [53]. Reaction mixture (200 μl) consisted of 10 μl of dilute enzyme extract and 100 μl of 0.1 M phosphate buffer (pH 7) and 60 μl of water. The reaction was initiated by adding 30 μl of 75 mM H_2_O_2_. A decrease in absorbance at 240 nm was observed every 30 s for over 3 min with UV- visible spectrophotometer.

### Sequence analysis of Nagina 22 and IR 64 SOD, APX, GR and CAT genes

The SOD, APX, GR, and CAT gene CDS and protein sequences from Nagina 22 and IR 64 were retrieved from the Manually Curated Database of Rice Proteins [54]. Nagina 22 sequences were aligned to IR 64 sequences using CLUSTALW and non-synonymous and synonymous single nucleotide polymorphisms (SNPs) were identified using BioEdit v7.2.5 software [55]. I-mutant3 [56] server was used to predict the effect of identified SNPs on protein stability. Network analysis in RiceNet v2 [57] was performed using ‘gene prioritization based on network direct neighborhood’ option, and viewed using Cytoscape v3.3.0 [58] for SOD, APX, GR and CAT genes.

### Data analysis and visualization

Majority of the data analysis was done in an open source R package [59] for statistical computing. Multivariate exploratory data analysis was done in FactomineR [60], ggbiplot [61], corrplot [62] and, Hmisc [63]. PerformanceAnalytics [64] was also used for data analysis and visualization. ANOVA for the enzyme assay was performed using the OP STAT web server (http://14.139.232.166/opstat/default.asp).

## Results

### Population performance under irrigated conditions and drought stress conditions at the reproductive stage

Summary statistics for the agronomical and physiological attributes measured in parents, the entire RIL population, extreme RILs identified, and the three bulks namely, the tolerant, sensitive and random bulks under both the irrigated condition (IC) and reproductive stage drought stress (RS) condition during the two cropping seasons in 2011 and 2014 are presented in Tables 1 and 2. Significant differences were observed between the treatments across the traits, especially for plant height, number of spikelets/panicle, spikelet sterility and single plant yield in the parents and in the entire population. Chlorophyll content (Table 2) measured as SPAD value, was better in IR64 under both the treatment conditions compared to Nagina 22. Tiller number (TN) showed variation in the tolerant parent and the tolerant bulk between IC and RS. Conversely, neither the sensitive parent nor the SB showed any difference in TN between IC and RS. There was an increase in the coefficient of variation (CV) for plant height, panicle length and single plant yield (SPY) under RS compared to IC in both years. The mean of the 41 extreme RILs identified was nearly identical to the population mean (Table 2), across all of the traits; moreover, there was an increase in the variation, suggesting that the identification of the extreme lines was robust. Although the extreme lines were identified based only on SS, the other traits also followed the same trend presumably because of the in-built trait correlations. This trend was more evident in single plant yield and SSI (Tables 1 and 2), perhaps because the trait correlations for these traits are more robust. Interestingly, the random bulk identified, consisting of just eight lines, had a mean identical to the population of 281 lines across all the eight traits, except for number of spikelets/panicle. Overall, the morphological observations collectively established the differential response of the parents and RILs in the mapping population for drought stress, reflecting their diverse genetic background and the robust relationship between the target trait and the stress. The differential performance of bulks as well as the parents with respect to SS under the IC and RS suggested that they could be used as a background to study genetical genomics.

**Table 1:** Descriptive statistics of agronomical and physiological parameters recorded in Nagina 22/IR 64 RIL population and bulks in 2011

	Plant height		Panicle length		Tiller number		Spikelets/panicle		Spikelet sterility		Single plant yield		SSI
	IC	RS	IC	RS	IC	RS	IC	RS	IC	RS	IC	RS	
Nagina 22	118.5	106.2	22.1	22.2	11.2	9.7	124.5	109.2	6.7	38	16.5	6.5	0.9
IR 64	88.9	75.53	24.2	23.89	11	11	113.8	108.4	7.5	88	19.2	2.5	1.4
Population													
Range	69.3–163.3	53–151	18.7–31.3	17–31	5.7–24.3	4.7–20.7	54.3–279.3	50–198.5	0.97–74.	7.4–99.7	5.8–48.7	2.2–24.4	0.2–1.6
Mean	129.67	104.1	24.49	24.08	12.47	10.6	129.19	97.24	12.38	47.3	22.18	9.03	0.96
SE mean	0.99	1.18	0.12	0.16	0.2	0.17	2.18	1.67	0.56	1.5	0.39	0.23	0.02
Var	273.18	346.88	4.08	6.47	11.32	7.14	1327	696.85	79.76	558.35	37.48	13.51	0.09
SD	16.53	18.62	2.02	2.54	3.36	2.67	36.43	26.4	8.93	23.63	6.12	3.68	0.29
CV	0.13	0.18	0.08	0.11	0.27	0.25	0.28	0.27	0.72	0.5	0.28	0.41	0.31
41 RILs													
Mean	125.3	102.4	24	23.6	12.6	11.1	122.3	94.5	10.05	53.21	22.91	9.21	0.95
Range	81.7–157.3	73–151	18.7–28.3	20.7–29.7	5.7–24.3	4.7–20.7	75–197	57.5–163.7	0.97–30.7	7.4–99	5.84–35.7	2.2–18	0.4–1.6
SE	2.94	3.3	0.29	0.36	0.62	0.43	4.75	3.93	1.21	5.96	1.08	0.66	0.05
CV	0.15	0.2	0.08	0.09	0.31	0.24	0.25	0.25	0.73	0.68	0.28	0.43	0.31
TB	123.88	105.49	24.29	24.88	13.04	11.08	122.25	113	8.82	22.16	22.13	9.82	0.87
SB	132.29	104.46	24.13	23.13	12.5	11.29	132.63	90.44	7.43	85.87	21.52	6.12	1.18
RB	127.46	98.71	24.75	23.96	13.59	10.75	128.43	92.66	15.12	64.84	23.6	9.25	0.97

*SSI* Spikelet sterility index, *IC* Irrigated control, *RS* Reproductive stage drought stress, *TB* Tolerant bulk, *SB* Susceptible bulk, *RB* Random bulk, *SE* Standard Error, *CI* Confidence interval, *SD* Standard deviation, *CV* Coefficient of variation

**Table 2:** Descriptive statistics of agronomical and physiological parameters recorded in Nagina 22/IR 64 RIL population and bulks in 2014

	Plant height		Panicle length		Tiller number		SPAD		Spikelets/panicle		Spikelet sterility		Single plant yield		SSI
	IC	RS	IC	RS	IC	RS	IC	RS	IC	RS	IC	RS	IC	RS	
Nagina 22	113.8	94.9	21.5	20.1	10	8.2	37.3	33.7	164.5	134	6.2	24	14.2	12.5	0.1
IR 64	90.1	63.7	23.5	20.1	8.7	8.3	41	36.7	156	119	8.1	91	17.2	1.6	1
Population															
Range	71.3–161	49–133	19.5–29.5	14.7–87.7	3.8–17.3	3.7–12.3	29.3–54.4	28–47.8	84.3–428.3	13.7–247.3	4.9–32.5	20.1–99.8	1.2–52.9	0.01–10.6	0.1–1.1
Mean	124.9	90.54	24.35	22.01	8.03	6.97	37.31	35.12	168.86	85.52	17.96	85.76	14.69	1.56	0.96
SE mean	0.93	0.92	0.12	0.38	0.11	0.09	0.21	0.17	2.71	1.84	0.3	1.01	0.55	0.1	0.01
Var	242.83	210.7	4.2	35.54	3.55	2.21	12.95	6.82	2068.34	843.38	22.31	254.24	74.31	2.57	0.04
SD	15.58	14.52	2.05	5.96	1.88	1.49	3.6	2.61	45.48	29.04	4.72	15.94	8.62	1.6	0.19
CV	0.12	0.16	0.08	0.27	0.23	0.21	0.1	0.07	0.27	0.34	0.26	0.19	0.59	1.03	0.2
41 RILs															
Mean	122.1	88.1	24	20.7	8.2	6.9	37.4	34.9	160.6	86.8	17.6	80.4	14.3	1.1	1
Range	84.2–148.7	51.3–119.7	21–26.5	16.3–25.7	5.5–12	4.7–9.3	29.3–46.1	30.3–42.5	103.7–255	42.3–181	9.2–32.5	30.5–99.6	1.2–26.5	0.04–4.03	0.21–1.12
SE	2.47	2.34	0.24	0.35	0.27	0.2	0.7	0.46	6.19	4.28	0.8	3.3	1.03	0.19	0.04
CV	0.13	0.16	0.06	0.1	0.21	0.18	0.12	0.08	0.25	0.3	0.28	0.25	0.43	1.03	0.22
TB	123.4	96.6	24	22.6	7.8	6.4	35.9	35.5	173.5	84.3	18.8	49.6	12.9	2.9	0.8
SB	118.1	84.8	23.8	20.1	8.4	7.8	37.2	34.1	163.3	87.8	16.9	91.6	15.4	0.4	1.1
RB	126	84.6	24.2	21	8.5	7	38.7	35	183.9	101.4	19.8	85.5	16.9	0.7	1

*SSI* Spikelet sterility index, *IC* Irrigated control, *RS* Reproductive stage drought stress, *TB* Tolerant bulk, *SB* Susceptible bulk, *RB* Random bulk, *SE* Standard Error, *CI* Confidence interval, *SD* Standard deviation, *CV* Coefficient of variation

### Enzyme assay

**Fig. 1:**
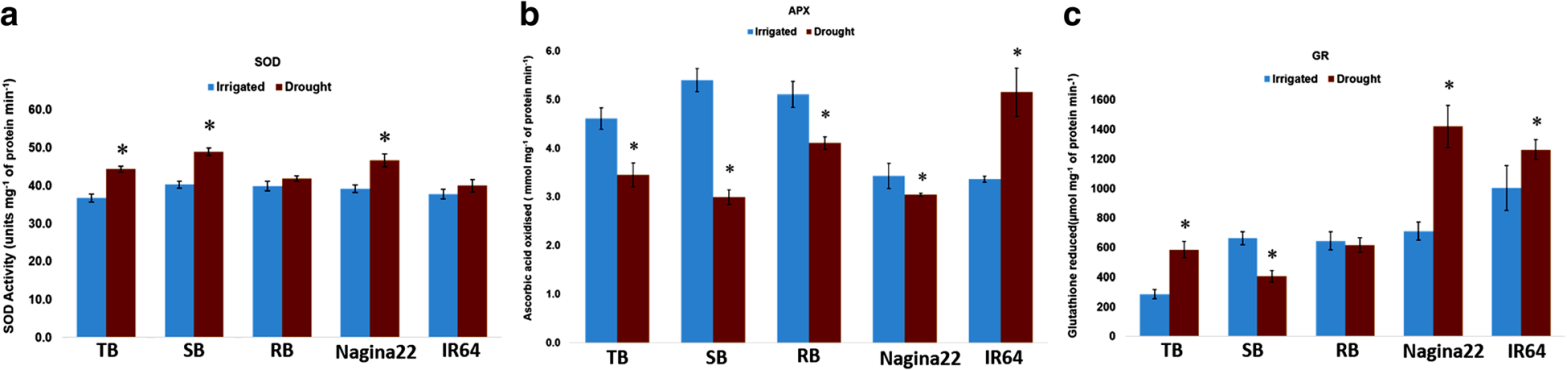
Enzyme assay results **a** TB, SB and Nagina 22 had more SOD activity under stress than control condition **b** Decreased APX activity under stress was observed across all bulks and Nagina 22 except IR 64 and **c** Nagina 22, IR 64 and TB showed more GR activity under stress as compared to control but not SB. TB: Tolerant bulk, SB: Susceptible bulk and RB: Random bulk. Vertical bars indicate ± SE. Asterisks (*) indicate significant difference (ANOVA, *P* ≤ 0.05) from control

The enzyme assays for all four enzymes (SOD, APX, GR, and CAT) were performed in the parents as well as in the constituent RILs of all the three bulks. However, CAT results were found to be error-prone despite repeating the experiment three independent times. Hence, the results of only three enzyme assays are presented in Fig. 1.

### SOD activity

Although, a general increase in SOD activity was observed in all the bulks and in the parental genotype under drought stress (Fig. 1a), the activity was more pronounced in TB, SB and Nagina 22 as compared to RB and IR 64 under stress situation. Thus, though distinct differences in SOD activity could be found between the parents, the same was not reflected in the extreme bulks.

### APX activity

Ascorbate peroxidase activity significantly decreased (Fig. 1b) across all the three bulks selected from the RIL population as well as in the drought tolerant parent, Nagina 22, under RS compared to IC. The quantum of decrease in activity was the least in Nagina 22 compared to SB and RB. However, in the other parent, IR 64, APX activity significantly increased under RS compared to IC. This could be because either APX is not a key player in OSM under drought stress or because IR 64 may have the better allele(s) for one or more of the APX genes.

### GR activity

Although GR activity was found to be enhanced in TB and in both of the parents under RS (Fig. 1c), the quantum of increase was much higher in the tolerant parent followed by TB. The other extreme bulk, SB showed a decreased activity under stress, whereas in RB, the difference in activity under IC and RS was negligible. Thus, GR was the only enzyme that had the expected activity in the contrasting parents and their extreme bulks.

The overall results of the enzyme assays though showed that different parents had better activity for different enzymes under stress, b﻿y comparing the quantum of ROS activity, Nagina 22 was superior to IR 64, at least for GR and SOD. The direction and quantum of expression in the extreme bulks established that there is a clear and robust correlation between the target trait SS and the GR activity in oxidative stress management under drought stress.

### Principal component analysis and linear correlations

**Fig. 2:**
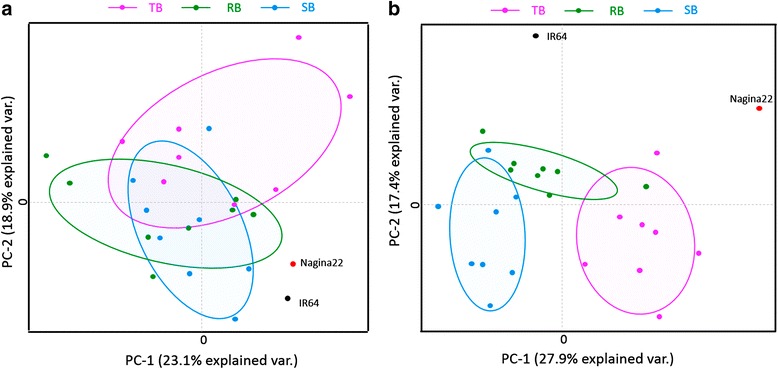
Individual factor map biplots of the morpho-physiological and enzyme assay as obtained from 2014 Kharif data. **a** Nagina 22 and IR 64 showed similar variation and were in the same quadrant. TB, SB and RB were also found to be overlapping under IC. **b** TB, SB and RB got differentiated into distinct groups under RS

**Fig. 3:**
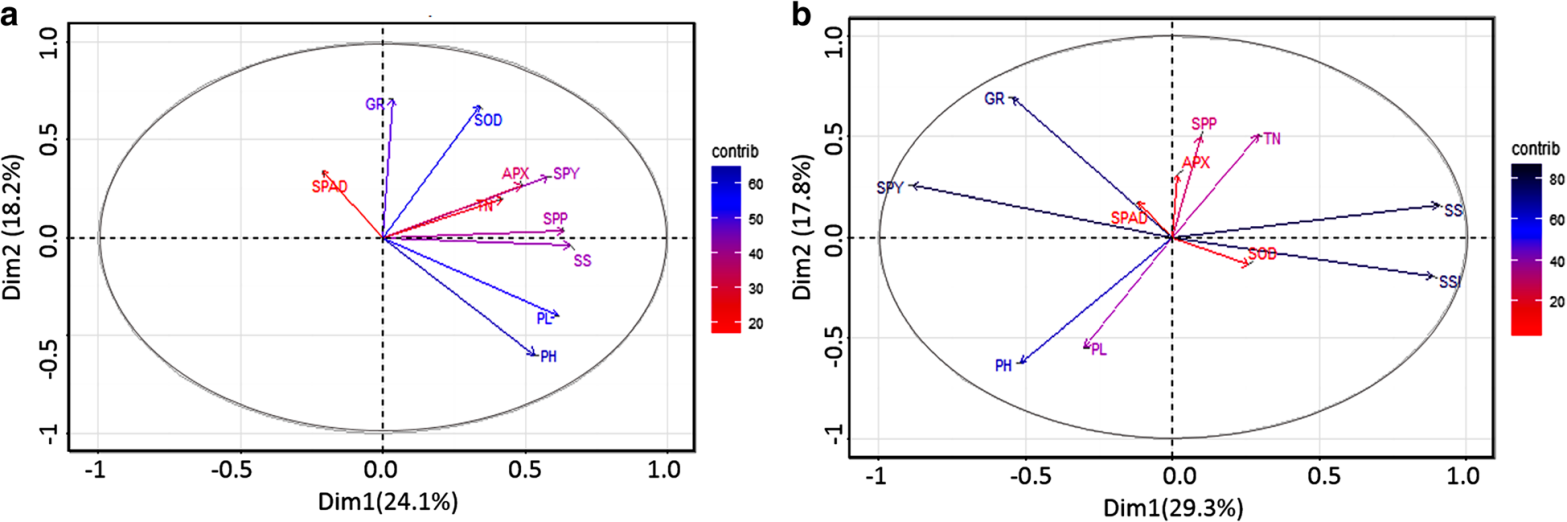
Principal component analysis of the morpho-physiological and enzyme assay as obtained from 2014 Kharif data **a** Under IC, tiller number (TN), and the enzymes, GR and SOD were found to be interacting and associated to PC-1 **b** SOD, APX and GR were found to be associated with SSI, SPP and SPAD respectively, under RS

**Fig. 4:**
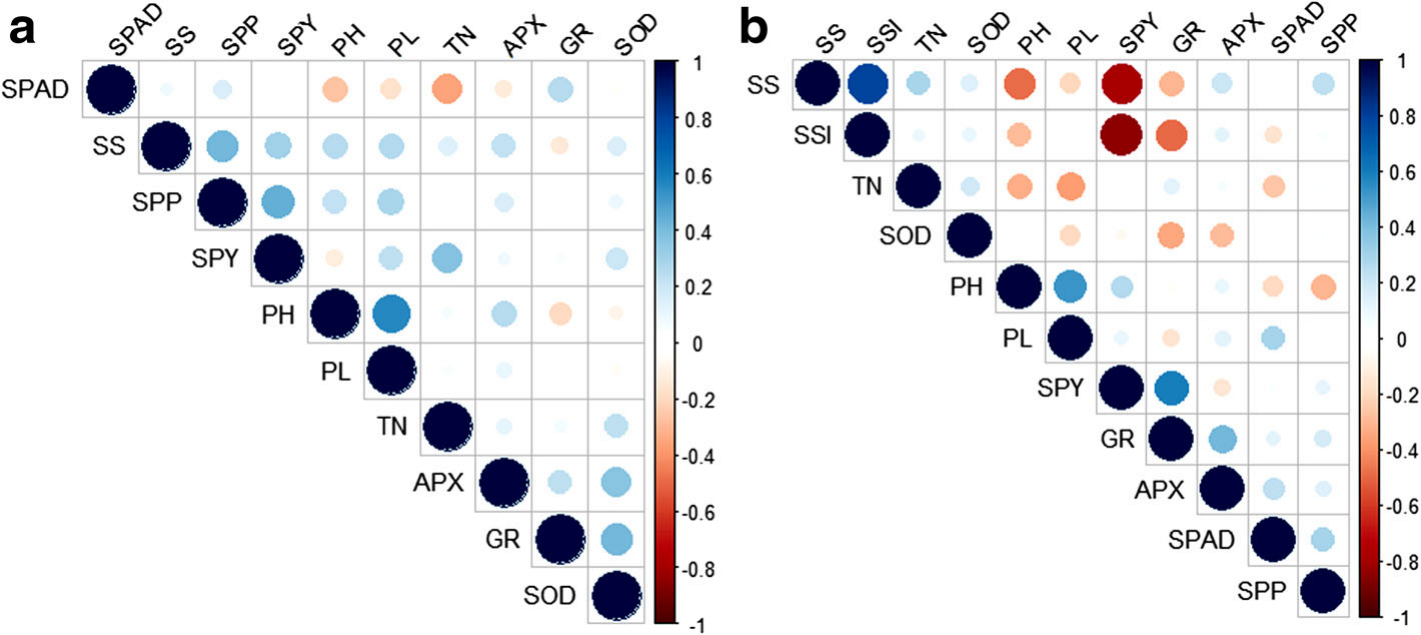
Correlogram of morpho-physio and enzymatic data **a** Irrigated control (IC): SOD, APX and GR were positively interacting with each other and **b** Reproductive stage drought stress (RS): SOD was negatively interacting with APX and GR while APX and GR were positively associated with each other

Since enzyme assay are the cumulative effect of multiple genes (as well as isoenzyme forms) that encode the enzymes in the genome, we undertook multivariate analysis. The multiple morphological and biochemical observations analysed for their possible correlated attributes by principal component analysis (PCA) and correlation analysis were expected to clarify these relationships because the observation of individual plants could be considered instead of the mean of the bulked RILs. Dimensionality of the data was revealed by an individual factor map (Fig. 2) which showed that the parents, TB, SB and RB had differentiated into distinct groups under RS compared to IC. Under RS, there was absolutely no overlap between the extreme bulks, whereas the random bulk was distributed across the extreme bulks thus, validating the suitability of the material under study. To capture the variability and interactions present in the morphological, physiological, and biochemical traits under different treatments, a variable factor map (Fig. 3) and correlogram (Fig. 4) were constructed. The first two principal components captured 24.1 % and 18.2 % of the variation under IC and 29.3 % and 17.8 % under RS, respectively. Under IC, TN, SPY, and the enzymes GR and SOD were found to interact and were highly associated to PC-1 while SPP and SS were associated with PC-2. This is consistent with our observation that the performance of TB and Nagina 22 was similar for TN, GR, and SOD (Tables 1, 2 and Fig. 1a and c). Under drought stress, the variability in APX, SPP, TN and SPAD were primarily captured by PC-1, whereas PC-2 explained the interactions for SOD and SSI. This suggests that APX is also an important component under drought stress, although Nagina 22, TB, and SB did not show any difference between the treatments (Fig. 1b). Thus, the multivariate analyses more clearly resolved the interactions between the enzyme assays and morphological traits under IC and RS.

Linear trait correlations including the enzyme assay from the parents and the RILs constituting the three bulks (Fig. 4) and the entire population were analysed individually (Additional file 1: Figure S1) under the two treatments. Since only the extreme RIL set, consisting of 26 individuals including the parents, had enzyme data, enzyme-morphological correlations were calculated usin﻿g this data set instead of the entire populatio﻿n under IC and RS. Correlations among OSM enzymes revealed that all three had significant and positive correlations under IC (GR-APX was not statistically significant), while under RS, GR had a significant and positive correlation with APX (Fig. 4a and b and Additional file 2: Table S1). However, SOD was negatively correlated with both APX and GR under RS. This could be the reason why the extreme bulks had the expected differences for GR and APX enzyme assay but not for SOD (Fig. 1). Among the enzyme-morphological correlations, only the correlation between SPY and GR was significant and positive under RS; and GR was significantly correlated with APX and SOD, albeit in the opposite direction. APX and SPAD readings were positively correlated under RS while negatively so under IC. Interestingly, under IC, none of the 21 pairs of correlations was significant between the OSM enzymes and the morphological traits. This clearly shows that the OSM pathway is significant only under drought stress and not under well-managed conditions. The target trait, SS also showed a robust correlation with the major productivity traits, SPY and SSI (Fig. 4a and b). Correlation analysis in IC and RS done in the population clearly defined the associations among the agronomic traits (Additional file 1: Figures S1a and S1b). For instance, under irrigated control conditions, PH was positively correlated to PL, SS, and SPP, and inversely related to SPY. Similarly, TN and SS were positively correlated under IC. However the hierarchy and the direction of correlation completely changed under RS. A positive relationship was observed between SPY, PH, SS and SPAD, negative correlation was observed between SPY and SSI under RS (Additional file 1: Figure S1b).

### SNPs and corresponding changes in proteins encoded by OSM genes in the parents

**Fig. 5:**
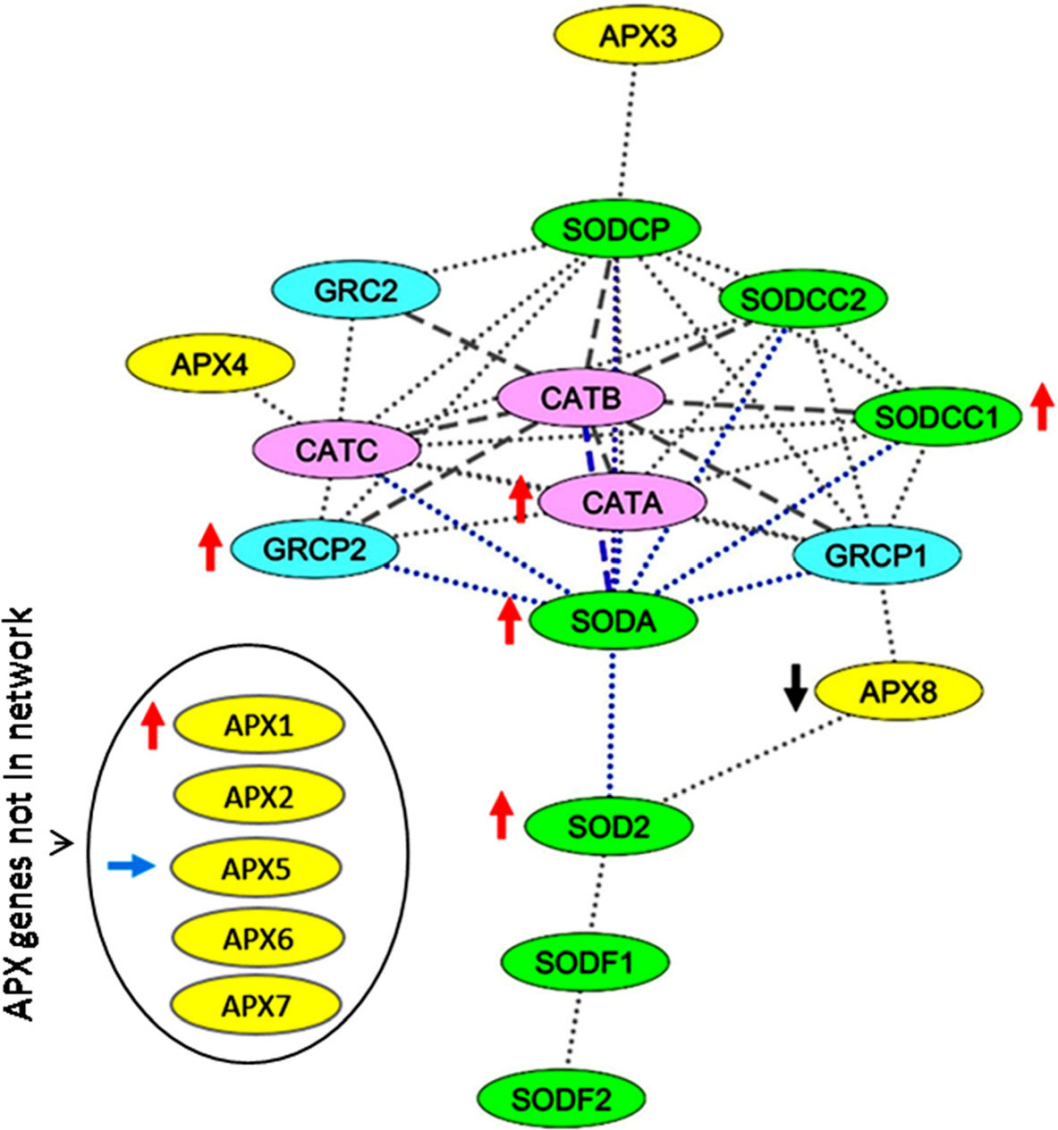
Interaction network of SOD, APX and GR and CAT genes in *Oryza sativa* ssp. *Japonica.* Nagina 22 has more thermostable SOD2, SODA, SODCC1, CATA and GRCP2 but not APX8 as compared to IR 64*.* All these stable isoforms of Nagina 22 directly interact with each other. Dashed and blue dotted line indicates network of CATB and SODA respectively. *Red*, *Black* and *Blue *arrow represents increased, decreased and neutral change in Nagina 22 protein stability as compared to IR 64

There was a total of 21 OSM genes in the rice genome, of which 7, 8, 3 and 3 code for SOD, APX, GR, and CAT, respectively. To investigate allelic differences in the multigenic ROS enzymes (allozymes) between the parents, SOD, APX, GR, and CAT gene sequences (18 of 21 genes; for two SOD and one CAT encoding genes, the sequences were not available) for the parents were retrieved. Both the CDS and protein sequences of SOD, APX, GR, and CAT were compared. Comparison of the CDS revealed that one-third of the ROS genes (7/21; two each from SOD, APX, and GR, and one from CAT) had no SNPs. Interestingly, most of the SNPs found resulted in non-synonymous amino acid substitutions in the proteins, especially in SOD and GR genes, which accounted for eight out of nine changes. In case of APX encoding genes, 45 % of SNPs (5/11) resulted in synonymous substitutions. In *APX5*, SNP substitution resulted in a missense mutation; still the resulting amino acid substitution (isoleucine to aspargine) did not alter the stability of the protein. In another *APX* gene (*APX4*), there was a long deletion (61 bp) as well as a frame shift mutation caused by a single nucleotide insertion that resulted in a completely different protein in IR 64. Overall, these changes led to a more thermodynamically stable SOD, APX, GR, and CAT proteins in Nagina 22 (nine out of eleven cases; Table 3 and Fig. 5). In only one gene *APX8*, the IR 64 allele had a more stable APX protein. The unfavorable allele from Nagina 22 for *APX8* was probably inherited by most of the plants that constituted the RB, SB, and TB. That is why APX activity in all the three bulks was similar to the Nagina 22 parent (Fig. 1a). An interesting feature observed was the occurrence of two point mutations in the same codon at c.169, 171 positions in a SOD coding gene (*SODA*) in IR64, which is rare. This resulted in the acidic to basic amino acid substitution -p.D57Q mutation that, in turn, resulted in a decrease in protein stability in the drought sensitive parent IR 64. Furthermore, the mitochondrial target of this gene makes it an important candidate in this spikelet sterility study. A SNP found between the parents in *GRCP2* but none of the other GR genes, corresponded perfectly with their enzyme assay results (Table 3 and Fig. 1). Although we did not have the CAT enzyme data, no SNPs were identified in *CATB* whereas *CATA* had three SNPs, all giving rise to the superior Nagina 22 allele.

**Table 3:** SNPs identified between Nagina 22 and IR 64 in the OSM genes, SOD, APX, GR and CAT

Gene	Symbol^a^	Locus ID (Rice MSU.v.7)	SNP (CDS region)	Change in amino acid	Protein stability in Nagina 22	Function
SOD	*SOD2*	LOC_Os03g11960	c.49 G > C	p.A17P	Increases	Copper/zinc superoxide dismutase, putative, expressed
	*SODCC1*	LOC_Os03g22810	c.40 C > A	p.L14I	Increases	Copper/zinc superoxide dismutase, putative, expressed
			c.130 G > A	p.G44R	Increases	
			c.281 C > T	p.P94L	Increases	
	*SODA*	LOC_Os05g25850	c.169 C > G,	p.Q57D	Increases	Superoxide dismutase, mitochondrial precursor, putative, expressed
			c.171 G > C			
			c.228 T > C	Unchanged	-	
	*SODF1*	LOC_Os06g02500	NA	-	-	Superoxide dismutase, chloroplast, putative, expressed
	*SODF2*	LOC_Os06g05110	No SNP	-	-	Superoxide dismutase, chloroplast, putative, expressed
	*SODCC2*	LOC_Os07g46990	No SNP	-	-	Copper/zinc superoxide dismutase, putative, expressed
	*SODCP*	LOC_Os08g44770	NA	-	-	Copper/zinc superoxide dismutase, putative, expressed
GR	*GRC2*	LOC_Os02g56850	No SNP	-	-	Glutathione reductase, cytosolic, putative, expressed
	*GRCP1* ^b^	LOC_Os03g06740	No SNP	-	-	Glutathione reductase, chloroplast, putative, expressed
	*GRCP2* ^b^	LOC_Os10g28000	c.394 G > A	p.D132N	Increases	Glutathione reductase, chloroplast, putative, expressed
APX	*APX8*	LOC_Os02g34810	c.1129 C > G	p.P377A	Decreases	OsAPx8 - Thylakoid-bound Ascorbate Peroxidase encoding gene 5,8, expressed
			c.1179 A > T	Unchanged	-	
	*APX1*	LOC_Os03g17690	c.269 C > T	p.T90I	Increases	OsAPx1 - Cytosolic Ascorbate Peroxidase encoding gene 1–8, expressed
			c.645 T > A	Unchanged	-	
	*APX3*	LOC_Os04g14680	No SNP	-	-	Peroxisomal Ascorbate Peroxidase encoding gene 5,8, expressed
	*APX7*	LOC_Os04g35520	c.72 G > A	Unchanged	-	OsAPx7 - Stromal Ascorbate Peroxidase encoding gene 5,8, expressed
			c.244 T > G	Unchanged	-	
			c.245 T > C	p.S82V	Increases	
	*APX2*	LOC_Os07g49400	No SNP	-	-	OsAPx2 - Cytosolic Ascorbate Peroxidase encoding gene 4,5,6,8, expressed
	*APX4*	LOC_Os08g43560	c.670 G > _	Frameshift	-	OsAPx4 - Peroxisomal Ascorbate Peroxidase encoding gene 5,8,9, expressed
	*APX6*	LOC_Os12g07820	c.468 T > C	Unchanged	-	OsAPx6 - Stromal Ascorbate Peroxidase encoding gene 5,8, expressed
	*APX5*	LOC_Os12g07830	c.266 T > A	p.I89N	Neutral	OsAPx5 - Stromal Ascorbate Peroxidase encoding gene 5,8, expressed
CAT	*CATA*	LOC_Os02g02400	c.418 T > A	p.F140I	Increases	Catalase isozyme A, putative, expressed
			c.437 G > A;c.438 C > G	p.R146Q	Increases	
			c.438 G > C;c.438 C > G	p.G148A	Increases	
	*CATB*	LOC_Os03g03910	NA	-	-	Catalase domain containing protein, expressed
	*CATC*	LOC_Os06g51150	No SNP	-	-	Catalase isozyme B, putative, expressed

I-mutant server was used to predict the protein stability changes because of SNP. Nagina 22 amino acid sequence was compared with corresponding IR 64 amino acid sequence and thermal stability was predicted

^a^Symbols retrieved from Uniprot database, ^b^symbols given by authors for easy understanding, NA: Gene sequence not available

### Network analysis of genes involved in OSM in rice

Our enzyme studies and multi-factor analyses suggested that all of the three enzymes are important players with respect to SS under drought stress, and all the SNPs between the parents in OSM genes, except one, led to more stable proteins in the drought tolerant parent Nagina 22; therefore we carried out a functional network analysis of the genes involved in OSM in rice. An interaction analysis showed that while all of the SOD, GR, and CAT genes were present in the network, for APX only three out of the eight genes were present (Fig. 5 and Additional file 3: Table S2). *APX3, APX4,* and *APX8* were found to interact with *SODCP, CATC, SOD2,* and *GRCP1,* respectively. *APX8* had more stability in IR 64 compared to the drought tolerant parent Nagina 22. Whatever limited drought tolerance exhibited by IR 64 could be because of this *APX8* gene. In Nagina 22, *SODA* could bypass the less stable form *APX8* and directly interact with four more SOD genes, and the two GR genes present in the central network. Thus the lesser stable form *APX8,* does not negatively affect the drought tolerant parent in terms of its ultimate phenotype, SS or yield under RS. Of the 11 genes present in the central network, Nagina 22 had more stable proteins encoded by *GRCP2*, *SODA* and *SODCC1*, *APX4,* and *CATA* than IR 64; whereas, both parents shared the same allele for four more genes (one SOD, two GR, and one CAT; Fig. 5 and Table 3). For the two other genes present in the network (one SOD and CAT each), although we did not have the SNP data, the network analysis revealed that the CAT gene interacted with only *APX4,* which had the functional allele in Nagina 22 but not in IR 64 because of a frame-shift mutation. Thus, the differences in the enzyme activity in the parents and extreme bulks under drought stress (Fig. 1) could be explained by the SNP changes and resultant protein stability in the parents, as suggested by the network analysis of the genes involved in the OSM pathway, and the multivariate analyses.

## Discussion

Breeding for drought tolerance in rice through introgression of QTLs that govern yield under drought [65, 66] is still an open area of research, as the causal genes are not yet known. Hence, one of the major challenges that researchers face is to unravel the complex mechanisms of drought tolerance through more intensive and integrated studies in order to find the key players or machineries that can be effectively exploited for developing drought tolerant crops [67]. Oxidative stress management (OSM) is one such key machinery or component of cellular tolerance under drought stress in different crop plants [68, 69]. Since rice has abundant genomic resources, it is possible to design studies without the need to invest huge financial resources, just by effectively combining forward and reverse genetic approaches. In this study, we made use of a mapping population we developed for the identification of QTLs of components for drought tolerance in rice, as well as utilized the genomic resources available in the public domain. One of the parents, Nagina 22, is tolerant to drought and known to have superior OSM [28] and higher spikelet fertility [23, 24] under drought.

We chose to use enzyme activity assays rather than real time transcript expression analysis for three reasons: (1) enzyme assays are more cost effective; (2) enzyme activity is a cumulative measure of activity of multiple genes; and (3) enzyme assays are closer to the phenotype than the transcript profiles to the phenotype, and our hypothesis for this work was that the biochemical or physiological component of phenotypes for complex traits should be identified along with morphological phenotypes through a genomic route that would allow for the identification of functional polymorphisms, and establish genotype-phenotype associations unequivocally in a Mendelian population.

The demonstration of the effect of drought stress on yield component traits and OSM enzyme activity under field conditions is not well documented in the available literature, especially in mapping populations. To our knowledge, this is the first report wherein enzyme activities have been measured under drought conditions imposed in the field and in well phenotyped extreme individuals following the principle of bulked segregant analysis in a synthetic Mendelian population [70, 71]. We decided to carry out the enzyme assays in extreme bulks for two reasons: (1) it would be difficult to do the enzyme assay in parents and all of the 281 RILs from the mapping population because biochemical assays need to be completed in sufficient biological and technical replicate: and (2) our target trait is well defined. We added a third bulk, called a random bulk, to reflect the scenario in the rest of the population.

Water stress caused significant reduction in PH, chlorophyll content, SF, and SPY in the RIL population including Nagina 22, IR 64, and the bulks (Tables 1 and 2) compared to the irrigated control. Although there was an overall decrease in productivity related traits in the population, Nagina 22 and TB showed more tolerance than did IR 64 and SB under drought. A stress susceptibility index that is a measure of drought resistance [72] showed Nagina 22 and TB to be better performers under drought stress than IR 64 and SB. RB showed an intermediate tolerance to drought as expected.

Water deficit causes a decline in photosynthetic rate and a limitation in carbon dioxide fixation, resulting in the exposure of chloroplasts to excess excitation energy and leading to the enhanced production of ROS, especially O^2−^ and H_2_O_2_ [73]. Drought stress caused increased SOD activity across all genotypes (Fig. 1a) compared to the irrigated control, indicating that high levels of H_2_O_2_ production and successful imposition of drought stress in field conditions. Lower APX activities in all bulks and Nagina 22 but not IR 64 (Fig. 1b) was observed under drought compared to the control, clearly showing an exhausted ascorbate pool because of drought stress. Increased GR activity in Nagina 22, IR 64, and TB (Fig. 1c) indicated an increased demand for reduced glutathione to combat the oxidative stress induced by drought. Both APX and GR work together to maintain the ascorbate-glutathione cycle, and the differential regulation of APX and GR enzymes in Nagina 22, IR 64, and their bulks under drought and control conditions is evidence that when one of the components of antioxidant defence is limiting it can be compensated for by the regulation of other components [8], suggesting a common regulatory mechanism of OSM genes [74] in rice.

Despite the compensatory mechanism of OSM, the enzyme activity patterns matched well with SNPs found in the OSM enzyme coding genes and their protein stability analyses (Fig. 1 and Table 3). For SOD and GR, the tolerant parent had higher activity under stress when compared to the sensitive parent. This is consistent with the observation that for two of the three GR enzymes, the parents had the same allele and for third one, the tolerant parent had the superior allele. Thus, the parents were monogenic with respect to GR activity. This explains why the direction of enzyme activity was the same while the quantum was different between the parents. Interestingly, the TB and SB behaved as though they have accumulated either all the favourable alleles or unfavourable alleles, respectively (Fig. 1c). This may account for the positive and significant GR-trait correlations (Additional file 2: Table S1). Such behaviour of extreme bulks could not be explained by simply accounting for differences in GR, but are explained when considering other OSM loci. In case of *SOD*, for two of the seven genes, the parents had identical alleles, and for the rest of the *SOD* genes, the tolerant parent had the superior allele. Although, the parents behaved in accordance with this allelic composition, the extreme bulks were identical to each other (Fig. 1a) and this explains why the SOD-trait associations were not significant (Additional file 2: Table S1). Such a complex system is bound to arise when multiple genes govern a target trait. Still, our network analysis along with the SNP analysis clearly showed the cross-talk among SOD, APX, GR, and CAT enzymes and for most of the key players (9/11) that are central to this interaction, the tolerant parent had the favourable alleles. Further expression analyses of all OSM enzyme encoding genes during RS and IC with leaf and root samples might suggest other clues on the difference in drought stress response between Nagina 22 and IR64. However, improving OSM is only one of the multiple factors that govern drought tolerance, especially SF under drought. Recently, the *OsCPK9* gene in rice, which belongs to the group III-b CDPK family, has been demonstrated to be involved in controlling SF under drought stress [25]. In RNA interference studies, *OsHXK10* has also been implicated in spikelet fertility by its role in pollen germination and anther dehiscence [75].

For most of the work on drought tolerance breeding in rice, a Nagina 22 x IR 64 population is available, and now markers can be designed based on the SNP information from our study, and used for marker-assisted selection of superior alleles of OSM genes. An ideal drought tolerant cultivar shall possess all of the superior alleles known thus far from the literature and efforts must continue to identify the yet unknown partners.

## Conclusions

The present study was carried out to unravel the oxidative stress management in rice under moisture deficit stress and associate the morphological, biochemical and genomic information to yield-related traits using a RIL mapping population of Nagina 22 and IR 64. Nagina 22 had superior alleles for SOD (SOD2, SODCC1, SODA) and GR (GRCP2) rather than for APX, for which IR 64 had the superior allele (APX8) in terms of predicted protein stability. Gene network and SNP analysis revealed the superiority of Nagina 22 in terms of drought tolerance at reproductive stage.

## Additional files

Additional file 1: Figure S1. Correlogram of the morpho-physio traits of the entire RIL population (*Kharif* 2014) under a Irrigated control (IC), SPY is positively associated with SPAD and SPP and b Reproductive stage drought stress (RS), SPY is negatively interacting with SS and SSI while positively with SPAD. (TIF 3276 kb)
Additional file 2: Table S1. Linear correlation r (Pearson) among morpho- physiological and enzyme results in parents and bulks under a IC: Irrigated control b RS: Reproductive stage drought stress (2014 *Kharif* data). (DOCX 17 kb)
Additional file 3: Table S2. Evidence for interaction network (.xlx). The file contains all the necessary scores and loci interaction details generated using RiceNet v2. (XLSX 14 kb)

## Abbreviations

APX: Ascorbate peroxidase
CAT: Catalase
GR: Glutathione reductase
OSM: Oxidative stress management
RB: Random bulk
RIL: Recombinant inbred lines
ROS: Reactive oxygen species
SB: Susceptible bulk
SF: Spikelet fertility
SOD: Superoxide dismutase
SPY: Single plant yield
SSI: Stress Susceptibility index
TB: Tolerant bulk
TN: Tiller number
