# Supplementary figures and images for: Unraveling the molecular basis of oxidative stress management in a drought tolerant rice genotype Nagina 22
Source: BMC Genomics. 2016 Oct 4;17:774. doi: 10.1186/s12864-016-3131-2 (PMC5050613; doi:10.1186/s12864-016-3131-2)

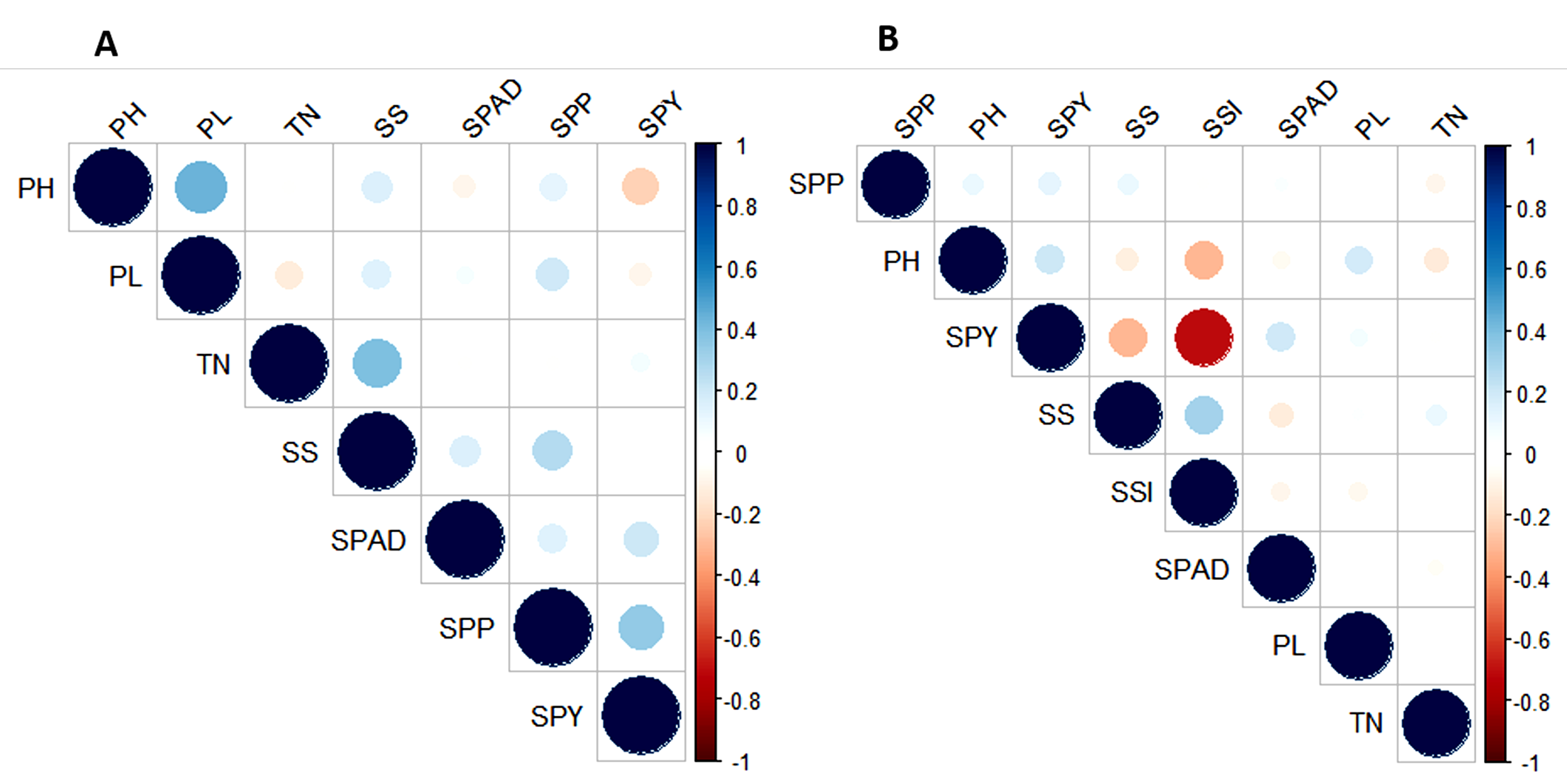

Supplement: Additional file 1: Figure S1. — Correlogram of the morpho-physio traits of the entire RIL population (Kharif 2014) under a Irrigated control (IC), SPY is positively associated with SPAD and SPP and b Reproductive stage drought stress (RS), SPY is negatively interacting with SS and SSI while positively with SPAD. (TIF 3276 kb) [file 12864_2016_3131_MOESM1_ESM.tif]
