# Supplementary material for: Unraveling the molecular basis of oxidative stress management in a drought tolerant rice genotype Nagina 22
Source: BMC Genomics. 2016 Oct 4;17:774. doi: 10.1186/s12864-016-3131-2 (PMC5050613; doi:10.1186/s12864-016-3131-2)
Supplement: Additional file 2: Table S1. — Linear correlation r (Pearson) among morpho- physiological and enzyme results in parents and bulks under a IC: Irrigated control b RS: Reproductive stage drought stress (2014 Kharif data). (DOCX 17 kb) [file 12864_2016_3131_MOESM2_ESM.docx]

**Additional file 2: Table S1:** Linear correlation t (Pearson) among morpho- physiological and enzyme results in parents and bulks under **a** IC: Irrigated control **b** RS: Reproductive stage drought stress (2014 Kharif data)

**A**

|  | PH | PL | TN | SPAD | SPP | SF | SPY | APX | GR | SOD |
| --- | --- | --- | --- | --- | --- | --- | --- | --- | --- | --- |
| PH | 1 |  |  |  |  |  |  |  |  |  |
| PL | 0.56* | 1 |  |  |  |  |  |  |  |  |
| TN | 0.06 | 0.05 | 1 |  |  |  |  |  |  |  |
| SPAD | -0.27 | -0.17 | -0.36* | 1 |  |  |  |  |  |  |
| SPP | 0.22 | 0.28 | 0.01 | 0.16 | 1 |  |  |  |  |  |
| SS | 0.25 | 0.26 | 0.15 | 0.08 | 0.41* | 1 |  |  |  |  |
| SPY | -0.12 | 0.23 | 0.37* | 0.02 | 0.44* | 0.3 | 1 |  |  |  |
| APX | 0.25 | 0.1 | 0.11 | -0.13 | 0.16 | 0.22 | 0.08 | 1 |  |  |
| GR | -0.2 | -0.01 | 0.07 | 0.25 | -0.01 | -0.14 | 0.05 | 0.23 | 1 |  |
| SOD | -0.09 | -0.06 | 0.23 | -0.05 | 0.09 | 0.16 | 0.2 | 0.36* | 0.41* | 1 |

**B**

|  | PH | PL | TN | SPAD | SPP | SF | SPY | SSI | APX | GR | SOD |
| --- | --- | --- | --- | --- | --- | --- | --- | --- | --- | --- | --- |
| PH | 1 |  |  |  |  |  |  |  |  |  |  |
| PL | 0.52* | 1 |  |  |  |  |  |  |  |  |  |
| TN | -0.33 | -0.38* | 1 |  |  |  |  |  |  |  |  |
| SPAD | -0.2 | 0.29 | -0.26 | 1 |  |  |  |  |  |  |  |
| SPP | -0.31 | -0.02 | 0.03 | 0.29 | 1 |  |  |  |  |  |  |
| SS | -0.49* | -0.21 | 0.28 | 0 | 0.23 | 1 |  |  |  |  |  |
| SPY | 0.26 | 0.1 | -0.03 | 0.04 | 0.11 | -0.78* | 1 |  |  |  |  |
| SSI | -0.29 | 0 | 0.09 | -0.16 | 0.04 | 0.79* | -0.85* | 1 |  |  |  |
| APX | 0.1 | 0.13 | 0.06 | 0.23 | 0.14 | 0.2 | -0.15 | 0.12 | 1 |  |  |
| GR | -0.05 | -0.16 | 0.13 | 0.12 | 0.17 | -0.31 | 0.59* | -0.5 | 0.41* | 1 |  |
| SOD | 0.02 | -0.2 | 0.18 | 0.02 | 0.04 | 0.15 | -0.07 | 0.1 | -0.29 | -0.35* | 1 |
